# Evaluation of Cerebral Blood Flow Dynamics in Transient Ischemic Attacks Patients with Fast Cine Phase Contrast Magnetic Resonance Angiography

**DOI:** 10.1155/2020/4097829

**Published:** 2020-04-13

**Authors:** Yuzhao Wang, Duo Gao, Huaijun Liu

**Affiliations:** Department of Medical Imaging, The Second Hospital of Hebei Medical University, Shijiazhuang 050000, China

## Abstract

Fast cine phase contrast magnetic resonance angiography (PC-MRA) has the potential to provide a quantitative measurement method for the diagnosis and treatment of cerebrovascular disease. To evaluation the changes of cerebral blood flow and the characteristics of artery lesion distribution in the patients of transient ischemic attacks (TIA). In all, 98 normal subjects and 106 TIA patients who underwent MRI examination within 72 h after the last symptom onset including the DWI sequence to exclude acute cerebral infarction were enrolled. The blood flow of the cranial total, the area of the internal carotid artery and vertebral artery, the average velocity, and the average blood flow were obtained and compared in normal subjects and TIA group. Analysis of Variance (ANOVA), *t*-test, and Kruskal-Wallis test were used for statistical assessments. The total cerebral blood flow of the TIA group and normal control group was no significant statistical difference (*P* > 0.05). The total blood flow decreased with increasing age, and the TIA group was much lower than the control group. The blood flow of the right internal carotid artery in the TIA group had a significant difference compared with controls (*P* < 0.05). However, the same situation did not happen in both of the left internal carotid artery and vertebral artery. Phase contrast magnetic resonance imaging has the potential to evaluate the change of cerebral blood flow in TIA patients. The decrease in the total blood flow and the symptom onset of TIA is consistent. Phase contrast magnetic resonance imaging could provide guidance to the diagnosis of TIA.

## 1. Introduction

Transient ischemic attacks (TIA) represent a frequent neurovascular condition associated with a risk of stroke recurrence of 5% at 48 h and 10% at 3 months in the absence of specific management [1]. Acute specialized management of TIA patients can significantly reduce the risk of stroke and other related vascular events such as myocardial infarction and vascular death [2–4].

In recent years, some studies have shown that TIA and ischemic stroke shared the same pathophysiological mechanism, but the prognosis may vary depending on the severity. Intracranial atherosclerosis (ICAS) is an important cause of ischemic stroke, especially in black, Hispanic, and Asian populations, where ICAS accounts for up to 50% of all ischemic strokes or TIAs [5–8]. Therefore, evaluation of cerebral blood flow dynamics in TIAs is essential.

The stationary proton will not produce phase displacement in the equilibrium gradient field, while the moving proton will produce phase displacement in the equilibrium gradient field. This is because the flow coding gradient has no effect on the stationary proton, and the stationary proton gets the same signal after two image acquisition. However, the phase displacement of the flowing protons is generated by the accumulation of two different positive and negative gradients between the two gradients, and this phase displacement is proportional to the movement of the flowing protons in the direction of the flow code. We subtract these two images, and we get an image of just the protons flowing. This phase change is the key of phase-sensitive flow imaging technology [9].

PC-MRA method, therefore, is to put MR technology for liquid phase displacement sensitivity combined with heart switch control technology, make the flow of the liquid phase displacement time combined with rate, thus further get all about the flow of the liquid phase, amplitude, waveform, the relationship between flow rate, and time of quantitative data of MR imaging technology; this technology has realized the MR of liquid flow rate and flow velocity of quantitative analysis. PC-MRA 2D Q-flow sequence is exactly based on this basic principle, so as to achieve MR flow-oriented quantitative research.

Moran [10] firstly proposed the MR quantitative study of blood flow in 1982. He utilized the principle of bipolar gradient phase generated between stationary tissue and fluid to detect the blood flow. Whereafter, Pernicone et al. [11] used phase contrast MRA to determine the anatomy and flow direction of the blood vessels, but they could not quantitatively analyze the blood flow. Later, Enzmann et al. [12] measured the velocity and flow rate of different vessels with phase contrast cine method, and then compared with the results of Transcranial Doppler (TCD) examination, it was found that there were some differences between MR measurement and TCD detection. The results showed that the maximum peak was poorly correlated. Fast cine phase contrast magnetic resonance angiography (PC-MRA) is a magnetic resonance imaging technique that has the ability to characterize velocity-time curves in the human coronary arteries with data acquisition. Fast cine phase contrast magnetic resonance angiography exploits the fact that moving spins accumulate different transverse phase when moving in the direction of a magnetic field gradient. The phase shift is proportional to velocity, and the proportionality constant is easily controlled. As a result, these techniques are ideally suited to quantitative application [13].

Hence, in this study we aimed at investigating the changes of cerebral blood flow and the characteristics of artery lesion distribution in both healthy subjects and subjects with TIA using fast cine PC-MRA.

## 2. Materials and Methods

### 2.1. Patients

This study was approved by the local Ethics Committee. Between May 2015 and June 2016, normal subjects and TIA patients were prospectively enrolled after written informed consent was obtained.

The inclusion criteria for healthy subjects included (1) no history of cerebrovascular disease; (2) no brain parenchyma lesions were detected by conventional MRI scan; (3) no vascular stenosis, occlusion, aneurysm, arterial dissection, or vascular malformation were detected by MRA; (4) no neurological symptom and signs; and (5) good image quality and complete data. The following conditions were excluded: severe hepatic and renal dysfunction; brain tumor, brain hemorrhage, and brain trauma; concurrent infection; mental disorders; and hematological system diseases.

For subjects with TIA, the inclusion criteria included (1) the principles for the diagnosis of TIA refer to the updated Chinese expert consensus on a transient ischemic attack; (2) no signs of acute ischemic stroke were detected by conventional MRI scan; (3) admission in 72 h from onset; and (4) no history of stroke.

The exclusion criteria included (1) no transient neurological deficits caused by hypoglycemia, epilepsy, and migraine; (2) transient global amnesia; (3) a history of stroke in 3 months; (4) brain tumor, brain hemorrhage, and brain trauma; (5) mental disorders; and (6) no cooperative or unable to undergo MR examination.

Overall, 89 healthy subjects (seven females and six males, age range 43–71 years) served as control participants and 106 subjects with TIA (seven females and six males, age range 40–75 years) were included in analysis.

### 2.2. MRI Acquisition Protocol

All subjects underwent brain MRI with a 3.0T MR scanner (3.0T Philips MR Systems Achieva, Best, Netherlands) equipped with a standard head coil. The standardized MR protocol included T1-weighted imaging, T2-weighted imaging, DWI, TOF-MRA, and Q-Flow PC-MRA. T1WI sequence parameters included slice thickness, 6.5 mm; slice gap, 1.3 mm; inversion time (TI), 900 ms; and TR/TE, 2000/20 ms. The parameters of T2WI and T2 FLAIR sequence: slice thickness and gap were consistent with the T1WI sequence; T2WI sequence parameters included TR = 5000 ms, TE = 120 ms; T2-FLAIR sequence parameters included TI, 2300 ms; TR/TE, 10000/120 ms. DWI was collected using single-shot echo-planar imaging with *b* values of 0 and 1000 s/mm2. repetition time (TR)/echo time (TE) = 2400/104 msec; field of view (FOV), 22 cm; matrix, 128 × 128; section thickness, 5 mm; and intersection gap 0 mm; number of slices, 17–18. 2D Q-Flow sequence was used to quantitatively detect the intracranial cerebral blood flow. The image of vascular localization was obtained based on the 3D TOF-MRA scanning, and the 2D Q-Flow scanning was performed perpendicular to the target vessel. 2D Q-Flow sequence parameters included 2D Q-Flow sequence scanning parameters: number of excitation (NEX) = 2, slice thickness = 4.0 mm, spacing = 0 mm, matrix = 512 × 256, field of view = 240 mm x 240 mm, reverse angle = 20°, bandwidth = 32. A phase change curve was obtained using peripheral gating, phaseless folding, respiratory compensation, and flow compensation techniques. The total of Phase = 16. The flow rate-coded Venc was set to 90 cm/s. Imaging time was approximately 15 to 20 minutes.

### 2.3. Image Analysis

#### 2.3.1. Image Reconstruction

3D TOF-MRA raw images were exported to the PHILIPS Extended MR WorkSpace 2.6.3.2 workstation. The raw images were reconstructed in three dimensions using the maximum intensity projection (MIP) method. The vascular images of the anterior and posterior cerebral circulation were obtained by postprocessing. Vessel morphology was recorded and the lumen diameter of the vessel layer corresponding to the 2D Q-Flow orientation was measured.

#### 2.3.2. Calculation of the Degree of Stenosis

The inner diameter of the internal carotid artery (ICA), anterior cerebral artery, middle cerebral artery, vertebral artery, basilar artery, and posterior cerebral artery was computed, respectively. The lumen at the narrowest place and the normal lumen at the distal of the stenosis were measured. According to the reduction of lumen diameter and the degree of signal loss, and the North American NASCET guidelines, the degree of stenosis is divided into normal, mild stenosis (0% < the extent of stenosis <50%), moderate stenosis (50% ≤ the extent of stenosis <75%), severe stenosis (75% ≤ the extent of stenosis <100%), and occlusion (completely signal loss).

Moderate stenosis of vessels or above associated with clinical symptoms was identified as a well-defined intracranial artery lesion. The tortuosity of the vessel was also classified as a mild stenosis group. If there are multiple degrees of stenosis in the same artery, the most severe stenosis is selected to calculate the degree of stenosis.

#### 2.3.3. Calculation of the Flow in the Lumen

Phase amplitude images and phase contrast images were obtained by 2D Q-Flow. The two sets of images were 30 images of different phases with the same cardiac cycle, which represents the function of flow velocity and time during the cardiac cycle. The images were exported to the PHILIPS Extended MR WorkSpace 2.6.3.2 workstation for postprocessing. Two experienced radiologists delineated the cross-sectional area of the region of interest (ROI) on the images. The diameter of the corresponding layer of the 3D TOF-MRA images is taken as a reference so that the cross-sectional area of ROI is the closest to the actual lumen. Then, the time-flow curve in a cardiac cycle would be obtained. The flow rate in the lumen per unit time can be obtained. The calculation formula is based on the flow rate (ml/min) = the flow rate (cm/s) × cross − sectional area (cm^2^) × 60 (s).

Firstly, a fluid model is designed, and it is found that the measured fluid signal strength has a good linear correlation with the velocity change, which indicates that the model can be used for the quantitative analysis of fluid.

The model lumen flow rate was set as 1.0 ml/s, 2.0 ml/s, 3.0 ml/s, 4.0 ml/s, and 5.0 ml/s, and the flow rate was measured according to the basic law of liquid flow *Q* = *v* r2, given that the inner diameter and flow rate of the lumen and the velocity of liquid flow in the lumen *v* = *Q*/*r*^2^ can be calculated. The measured velocity was positively correlated with the actual velocity (correlation coefficient = 0.98, *P* < 0.0001). Therefore, a cross-sectional area is a parameter to measure the flow rate.

#### 2.3.4. Statistical Analysis

SPSS Statistics (v. 22, IMB, Armonk, NY) was used for statistical analyses. Data distributions were tested with the Shapiro–Wilk W test. All data were expressed as mean ± standard deviation, except where noted. Mean flow velocity, peak flow rate, and flow rate of the bilateral internal carotid artery, A1 segment in anterior cerebral artery, M1 segment in middle cerebral artery, vertebral artery, basilar artery, and P1 segment in the posterior cerebral artery were analyzed with Student's *t* test. The flow rate and flow of intracranial artery between different genders were compared by the two sample *t* test. If the data for the two groups were nonnormally distributed or the variances were not equal, the nonparametric Kruskal-Wallis test was used. The statistical significance level was set at *P* < 0.05 for all analyses.

## 3. Results and Discussion

106 patients (56 male, 50 female) with TIA, ranging in age from 21 to 85 years (58.05 ± 19.10), were evaluated in MRI routine examination including MRI, DWI, and MRA within 72 hours after the last symptom. The control group included 50 qualified volunteers, including 26 males and 24 females in age from 28 to 71. Their demographic characteristics including mean ± standard deviation, age, body mass index (BMI), hypertension, diabetes mellitus, smoke, and Atrial fibrillation are listed in Table 1.

### 3.1. The Degree of Stenosis and Distribution Characteristics in Patients with TIA

The narrowness of varying degrees between the TIA group and the control group is shown in Table 2. There is no statistically significant difference between the incidence of mild stenosis in the TIA group and the control group. However, there was a statistically significant difference between the incidence of severe stenosis and the normal control group in the TIA group (*P* < 0.05).

**Figure 1:**
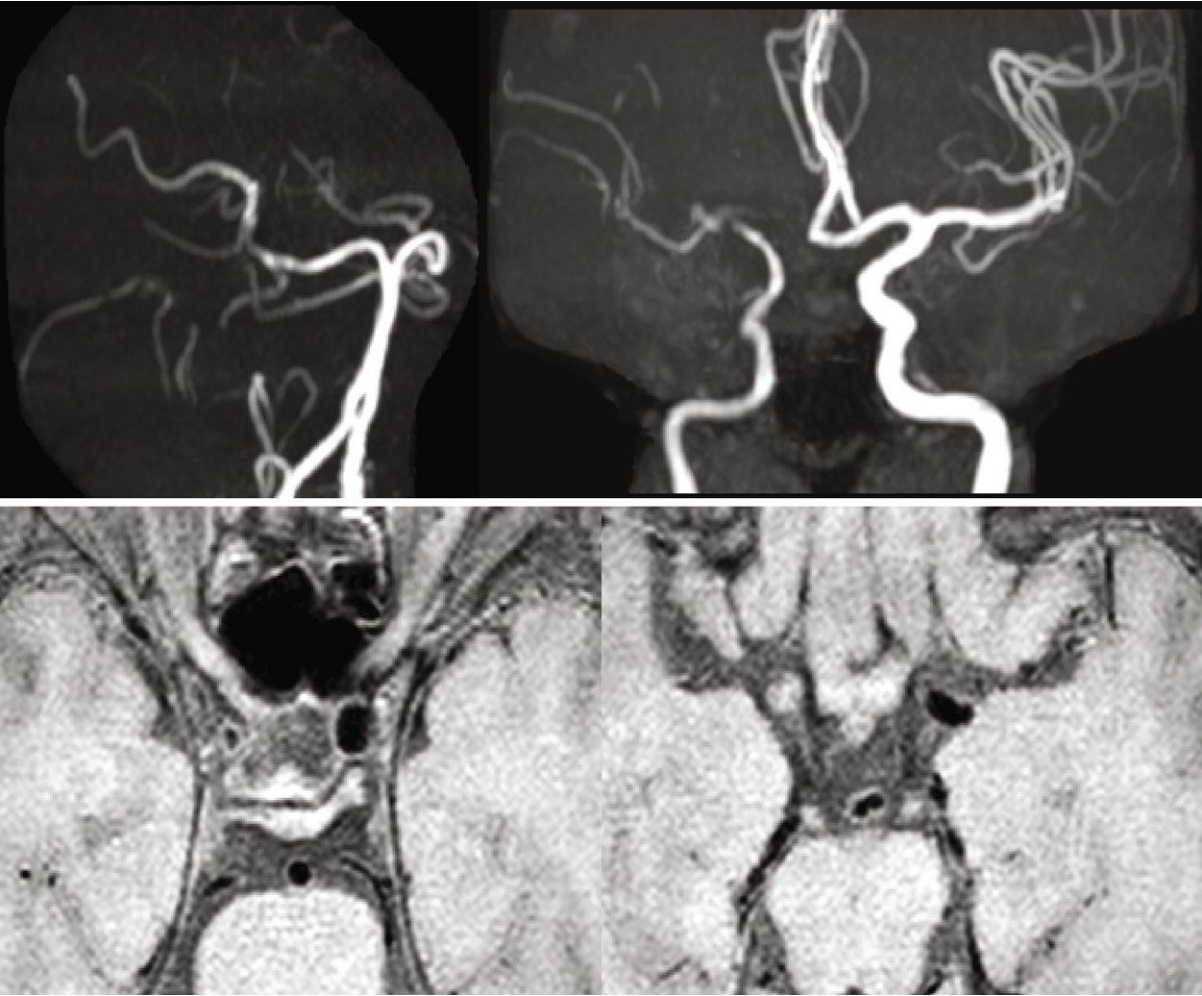
PC-MRA and TOF-MRA for different cerebrovascular stenosis.

**Figure 2:**
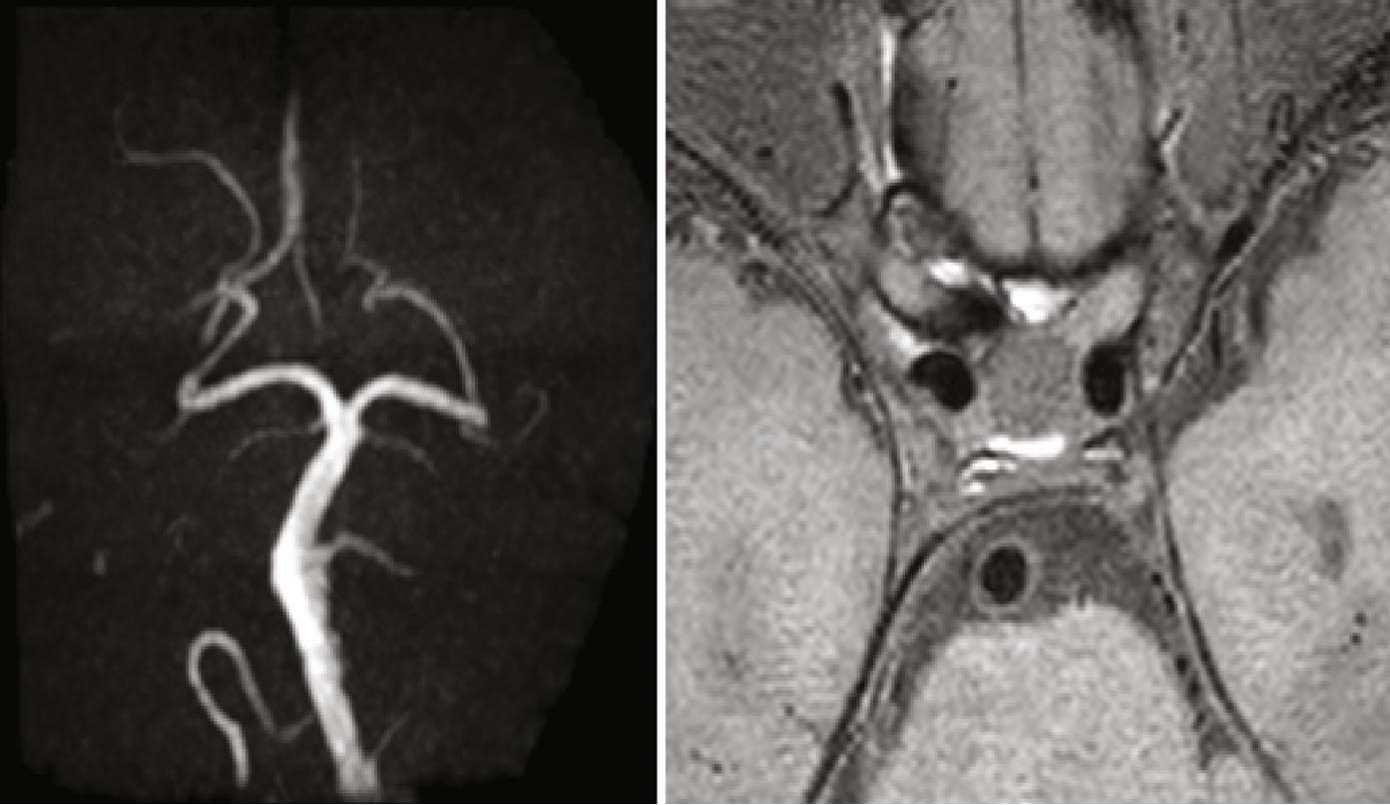
PC-MRA and TOF-MRA for BA mild inhomogeneous stenosis.

**Figure 3:**
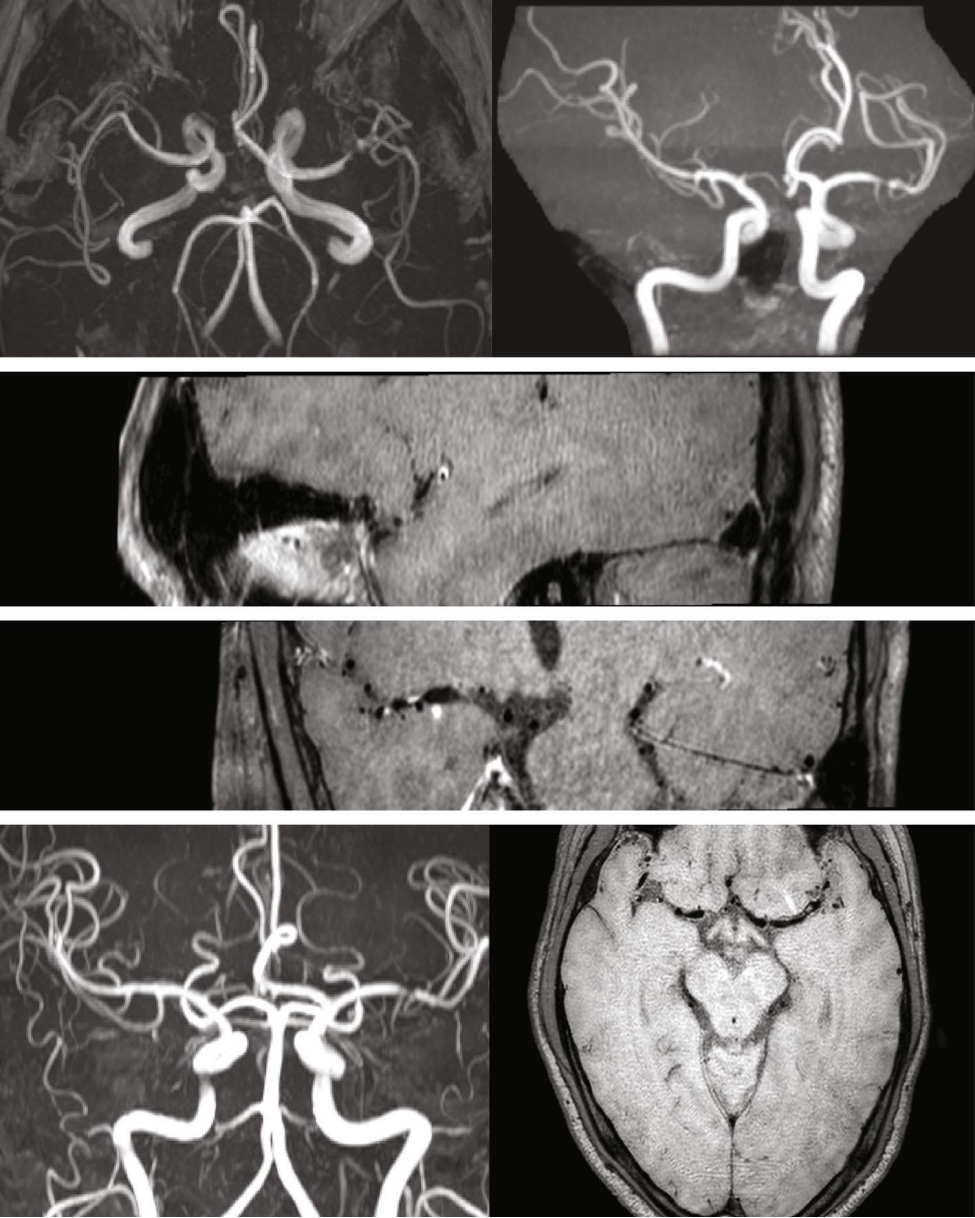
PC-MRA and TOF-MRA for MCA stenosis.

In the 106 TIA subjects, 69 had different degrees of vascular stenosis, including 19 with anterior cerebral artery stenosis, 21 with cerebral artery stenosis, 28 with internal carotid artery stenosis, and 17 with posterior cerebral artery stenosis; 23 patients with vertebral artery stenosis; and 12 patients with basilar artery stenosis. There are two sample demonstrated moderate middle cerebral artery stenosis and BA mild inhomogeneous stenosis using PC-MRA and TOF-MRA (Figures 1–3). There were only 26 cases involving one vessel stenosis, 29 cases with two vessels, and 14 patients with three or more vessel stenosis. There were no significant differences in different vascular stenosis groups (*P* > 0.05). There was also no significant difference between the cumulative one and two-vessel groups (*P* > 0.05), and there was a statistical difference between the one or two-vessel groups with the cumulative three or more vessel groups (*P* < 0.05). There was no significant difference in the degree of vascular stenosis between different gender groups (*P* > 0.05).

### 3.2. The Characteristics of Cerebral Flow in Patients with TIA

The main blood supply of the head is bilateral ICA and vertebral artery and basilar artery (V-BA). In this study, we consider the sum of bilateral ICA and V-BA blood flow as the whole cerebral flow to compare the TIA group and control group in total cerebral blood flow (Table 3). The results showed that there was no significant difference in the whole cerebral blood flow between the TIA group and the normal control group (*P* > 0.05).

The total blood flow decreased with age. The total blood flow in the TIA group was slightly lower than that in the control group, but there was no statistically significant difference (*P* > 0.05). The right internal carotid artery flow in the TIA group was significantly different from the control group (*P* < 0.05). The right internal carotid artery flow in the TIA group decreased compared with the control group. There was no significant difference in the left internal carotid artery and bilateral vertebral artery flow between the TIA group and the control group (Table 4).

### 3.3. The Relationship between the Occurrence of TIA and Atherosclerotic Lesions

Among the 106 patients with TIA, 62 patients had different degrees of atherosclerotic lesions, including 26 cases involving internal carotid artery, 22 cases involving only intracranial arteries, and 14 cases involving both internal carotid artery and intracranial artery. There were 21 cases of atherosclerotic lesions in 50 cases of the control group. 9 cases were involved in the internal carotid artery, only 8 cases involving the intracranial artery, and 4 cases involving both the internal carotid artery and intracranial artery. There was no statistically significant difference in the distribution of atherosclerotic lesions in the cranial neck between the TIA patients and the control group (*P* > 0.05).

### 3.4. The Relationship between the Occurrence of TIA and Vascular Morphology

The proportion of vascular dysplasia or vascular variability in the TIA group (38.7%) is slightly higher than the control group (33.9%), but there was no significant statistical difference (*P* > 0.05).

## 4. Conclusions

Most TIA patients have varying degrees of stenosis or occlusion of the supply artery. The risk of ischemic stroke in these TIA patients increased with some risk factors such as the degree, extent, or duration of vascular stenosis. Carotid, vertebral and intracranial atherosclerosis, wall thickening, unstable plaque formation, and others can lead to the stenosis of the vascular lumen. In this study, 58% of TIA patients were accompanied by atherosclerotic lesions of cranial and cervical supply arteries, mostly involving the bifurcation of the carotid artery and the internal carotid artery. In the TIA group, the blood supplement of ICA and V-BA that accompanied atherosclerotic lesions was significantly increased compared with the normal control group. Therefore, poor vascular development and atherosclerosis are likely to be one of the risk factors for TIA.

In this study, the 2D Q-flow PC-MRA method was used to compare the cerebral blood Flow of TIA patients and the normal control group. We analyzed that the symptoms of some TIA patients may be caused by microembolic embolization of the small distal supply artery, but there were no significant changes in total cerebral blood flow. Some studies have found that there is no significant difference between whole brain blood flow in patients with atherosclerosis and normal healthy group, which is consistent with the results of this study. Since the MR examination of all TIA patients in this study was conducted after the onset of symptoms, it could not fully show the vascular conditions at the onset of TIA. Therefore, it has certain limitations.

In conclusion, atherosclerotic disease and changes of vascular dysplasia or morphologic are likely to be the risk factors for TIA. Magnetic resonance phase contrast angiography provides more evidence for the diagnosis of transient ischemic attack, and it has a broad clinical application prospect.

## Figures and Tables

**Table 1:** Baseline characteristics of the TIA group and control group.

	TIA group (*n* = 106)	Control group (*n* = 50)	*T*/*χ*^2^	*P*
Age	58.05 ± 19.10	60.14 ± 12.20	25.61	0.44
Male	56 (0.53)	26 (0.52)	0.095	0.76
Hypertension	20 (0.19)	4 (0.08)	0.352	0.553
Diabetes mellitus	20 (0.19)	3 (0.06)	13.344	0.03
Smoke	54 (0.51)	14 (0.50)	1.835	0.176
Atrial fibrillation	19 (0.18)	3 (0.06)	20.448	0.02

**Table 2:** Narrowness of varying degrees between the TIA group and control group.

	TIA group (total 106)	Control group (total 50)	*P*
Nonstenosis	37	21	0.23
Mild stenosis	40	22	0.22
Moderate stenosis	20	7	0.15
Severe stenosis	9	0	0.03

**Table 3:** The blood flow of the internal carotid artery (ICA) and vertebral artery (VA) between the TIA group and control group.

	*Flow(ml/min)*		
	TIA group	Control group	*P*
ICA(L)	238 ± 63	242 ± 71	0.38
ICA(R)	261 ± 71	271 ± 79	0.45
VA(L)	67 ± 39	69 ± 45	0.54
VA(R)	59 ± 37	63 ± 39	0.26

**Table 4:** The blood flow of the internal carotid artery (ICA) and vertebral artery (VA) among different TIA group and control group.

	*Flow(ml/min)*			
	TIA group (ICA)	TIA group (V-BA)	Control group	*P*
ICA(L)	227 ± 67	221 ± 74	242 ± 71	0.09
ICA(R)	221 ± 76	272 ± 81	271 ± 79	0.038
VA(L)	76 ± 36	59 ± 47	69 ± 45	0.07
VA(R)	74 ± 37	61 ± 44	63 ± 39	0.12

## Data Availability

The data used to support the findings of this study are included within the article.
